# Continuous Journey Toward Polymer Applications

**DOI:** 10.3390/polym12020312

**Published:** 2020-02-04

**Authors:** Wei Min Huang

**Affiliations:** School of Mechanical and Aerospace Engineering; Nanyang Technological University, 50 Nanyang Avenue, Singapore 639798, Singapore; mwmhuang@ntu.edu.sg

In 2019, 498 papers were published under the section of “Polymer Applications” in *Polymers*, which covers a range of interesting topics. There were also 99 Special Issues that addressed some hot topics. 

While 3D printing, as a newly emerged technology, attracts attention from a lot of researchers as a method with which to explore new ways to fabricate polymeric devices with enhanced performance and/or new functions [1,2,3,4,5], sensors and actuators (including wearable/flexible electronic devices) still appear to be the major focus in polymer applications [1,5,6,7,8,9,10,11,12,13,14,15]. Polymers with special features/functions, such as stimulus-responsive and shape memory [16,17], self-healing [18,19,20,21,22], and assembly [23,24,25,26,27] are being continuously developed, along with analytical/numerical studies [28,29,30], in order to capture the fundamentals for efficient and/or optimized engineering design. Of course, the above mentioned are only parts of the topics of the published papers in 2019 under this section, which focuses on polymer applications. Continuous efforts are needed to investigate the relationships between polymer structures and their properties [22], to enhance filtration/separation [31,32], and to improve the performance of polymers via the concept of composites/nanocomposites [16,31]. Of course, biomedical engineering is currently a very important application field for polymers [2,33,34]. 

It is not possible to list all these wonderful papers, which are inspiring and helpful to the community of polymer applications. We appreciate these contributions, and hope that 2020 is another successful year for *Polymers* and this section.

## References

[1] Liu C., Huang N., Xu F., Tong J., Chen Z., Gui X., Fu Y., Lao C. (2018). 3D Printing Technologies for Flexible Tactile Sensors toward Wearable Electronics and Electronic Skin. Polymers.

[2] Chiu Y.-C., Shen Y.-F., Lee A.K.-X., Lin S.-H., Wu Y.-C., Chen Y.-W. (2019). 3D Printing of Amino Resin-based Photosensitive Materials on Multi-parameter Optimization Design for Vascular Engineering Applications. Polymers.

[3] Eom R.-I., Lee H., Lee Y. (2019). Evaluation of Thermal Properties of 3D Spacer Technical Materials in Cold Environments using 3D Printing Technology. Polymers.

[4] Park J.H., Lee J.R. (2019). Developing Fall-impact Protection Pad with 3D Mesh Curved Surface Structure using 3D Printing Technology. Polymers.

[5] Wang J., Liu Y., Su S., Wei J., Rahman S.E., Ning F., Christopher G., Cong W., Qiu J. (2019). Ultrasensitive Wearable Strain Sensors of 3D Printing Tough and Conductive Hydrogels. Polymers.

[6] Al-Halhouli A., Al-Ghussain L., El Bouri S., Liu H., Zheng D. (2019). Fabrication and Evaluation of a Novel Non-Invasive Stretchable and Wearable Respiratory Rate Sensor Based on Silver Nanoparticles Using Inkjet Printing Technology. Polymers.

[7] Yu S., Dong S., Jiao X., Li C., Chen D. (2019). Ultrathin Photonic Polymer Gel Films Templated by Non-Close-Packed Monolayer Colloidal Crystals to Enhance Colorimetric Sensing. Polymers.

[8] Park M., Yoo S., Bae Y., Kim S., Jeon M. (2019). Enhanced Stability and Driving Performance of GO⁻Ag-NW-based Ionic Electroactive Polymer Actuators with Triton X-100-PEDOT:PSS Nanofibrils. Polymers.

[9] Harjo M., Tamm T., Anbarjafari G., Kiefer R. (2019). Hardware and Software Development for Isotonic Strain and Isometric Stress Measurements of Linear Ionic Actuators. Polymers.

[10] Wang P., Wei S., Tong L., He X., Bai Y., Jia K., Liu X. (2019). An Immunosensor Based on Au-Ag Bimetallic NPs Patterned on a Thermal Resistant Flexible Polymer Substrate for In-Vitro Protein Detection. Polymers.

[11] Dong Q., Sun C., Chen F., Yang Z., Li R., Wang C., Luo C. (2019). Influence of Cyclodextrins on Thermosensitive and Fluorescent Properties of Pyrenyl-Containing PDMAA. Polymers.

[12] Park M.S., Meresa A.A., Kwon C.-M., Kim F.S. (2019). Selective Wet-Etching of Polymer/Fullerene Blend Films for Surface- and Nanoscale Morphology-Controlled Organic Transistors and Sensitivity-Enhanced Gas Sensors. Polymers.

[13] Tohluebaji N., Putson C., Muensit N. (2019). High Electromechanical Deformation Based on Structural Beta-Phase Content and Electrostrictive Properties of Electrospun Poly(vinylidene fluoride- hexafluoropropylene) Nanofibers. Polymers.

[14] Xiang C., Guo J., Sun R., Hinitt A., Helps T., Taghavi M., Rossiter J. (2019). Electroactive Textile Actuators for Breathability Control and Thermal Regulation Devices. Polymers.

[15] Martella D., Nocentini S., Antonioli D., Laus M., Wiersma D.S., Parmeggiani C. (2019). Opposite Self-Folding Behavior of Polymeric Photoresponsive Actuators Enabled by a Molecular Approach. Polymers.

[16] Wang L., Luo B., Wu D., Liu Y., Li L. (2019). Fabrication and Characterization of Thermal-responsive Biomimetic Small-scale Shape Memory Wood Composites with High Tensile Strength, High Anisotropy. Polymers.

[17] Frewin C.L., Ecker M., Joshi-Imre A., Kamgue J., Waddell J., Danda V.R., Stiller A.M., Voit W.E., Pancrazio J.J. (2019). Electrical Properties of Thiol-ene-based Shape Memory Polymers Intended for Flexible Electronics. Polymers.

[18] Qian Y., An X., Huang X., Pan X., Zhu J., Zhu X. (2019). Recyclable Self-Healing Polyurethane Cross-Linked by Alkyl Diselenide with Enhanced Mechanical Properties. Polymers.

[19] Wu B., Zhang Y., Yang D., Yang Y., Yu Q., Che L., Liu J. (2019). Self-Healing Anti-Atomic-Oxygen Phosphorus-Containing Polyimide Film via Molecular Level Incorporation of Nanocage Trisilanolphenyl POSS: Preparation and Characterization. Polymers.

[20] Naveed M., Rabnawaz M., Khan A., Tuhin M.O. (2019). Dual-Layer Approach toward Self-Healing and Self-Cleaning Polyurethane Thermosets. Polymers.

[21] Irigoyen M., Matxain J.M., Ruipérez F. (2019). Effect of Molecular Structure in the Chain Mobility of Dichalcogenide-Based Polymers with Self-Healing Capacity. Polymers.

[22] Li T., Zheng T., Han J., Liu Z., Guo Z.-X., Zhuang Z., Xu J., Guo B.-H., Guo A.B.-H. (2019). Effects of Diisocyanate Structure and Disulfide Chain Extender on Hard Segmental Packing and Self-Healing Property of Polyurea Elastomers. Polymers.

[23] Fauquignon M., Ibarboure E., Carlotti S., Brûlet A., Schmutz M., Le Meins J.-F. (2019). Large and Giant Unilamellar Vesicle(s) Obtained by Self-Assembly of Poly(dimethylsiloxane)-b-poly(ethylene oxide) Diblock Copolymers, Membrane Properties and Preliminary Investigation of Their Ability to Form Hybrid Polymer/Lipid Vesicles. Polymers.

[24] Jo G., Jung J., Chang M. (2019). Controlled Self-Assembly of Conjugated Polymers via a Solvent Vapor Pre-Treatment for Use in Organic Field-Effect Transistors. Polymers.

[25] Yu H.-C., Lin C.-H., Yang C.-I. (2019). Temperature-Controlled Assembly/Reassembly of Two Dicarboxylate-Based Three-Dimensional Co(II) Coordination Polymers with an Antiferromagnetic Metallic Layer and a Ferromagnetic Metallic Chain. Polymers.

[26] Ling W., Cheng X., Miao T., Zhang S., Zhang W., Zhu X. (2019). Synthesis and Photocontrolled Supramolecular Self-Assembly of Azobenzene-Functionalized Perylene Bisimide Derivatives. Polymers.

[27] Gu L., Xie M.-Y., Jin Y., He M., Xing X.-Y., Yu Y., Wu Q.-Y. (2019). Construction of Antifouling Membrane Surfaces through Layer-by-Layer Self-Assembly of Lignosulfonate and Polyethyleneimine. Polymers.

[28] Yang Z.X., He X.T., Jing H.X., Sun J.Y. (2019). A Multi-Parameter Perturbation Solution and Experimental Verification for Bending Problem of Piezoelectric Cantilever Beams. Polymers.

[29] Debeleac C., Nechita P., Nastac S. (2019). Computational Investigations on Soundproof Applications of Foam-Formed Cellulose Materials. Polymers.

[30] Lin J.-T., Liu H.-W., Chen K.-T., Cheng D.-C. (2019). Modeling the Optimal Conditions for Improved Efficacy and Crosslink Depth of Photo-Initiated Polymerization. Polymers.

[31] Xie Q., Zhang S., Ma H., Shao W., Gong X., Hong Z. (2018). A Novel Thin-Film Nanocomposite Nanofiltration Membrane by Incorporating 3D Hyperbranched Polymer Functionalized 2D Graphene Oxide. Polymers.

[32] Geng Z., Wang X., Jiang H., Zhang L., Chen Z., Feng Y., Geng W., Yang X., Huo M., Sun J. (2019). High-performance TiO2 nanotubes/poly (aryl ether sulfone) hybrid self-cleaning anti-fouling ultrafiltration membranes. Polymers.

[33] Gao T., Jiang M., Liu X., You G., Wang W., Sun Z., Ma A., Chen J. (2019). Patterned Polyvinyl Alcohol Hydrogel Dressings with Stem Cells Seeded for Wound Healing. Polymers.

[34] Aguilar L.E., Lee J.Y., Park C.H., Kim C.S. (2019). Biomedical Grade Stainless Steel Coating of Polycaffeic Acid via Combined Oxidative and Ultraviolet Light-Assisted Polymerization Process for Bioactive Implant Application. Polymers.

